# Superior survival with pediatric-style chemotherapy compared to myeloablative allogeneic hematopoietic cell transplantation in older adolescents and young adults with Ph-negative acute lymphoblastic leukemia in first complete remission: analysis from CALGB 10403 and the CIBMTR

**DOI:** 10.1038/s41375-021-01213-5

**Published:** 2021-03-30

**Authors:** Matthew J. Wieduwilt, Wendy Stock, Anjali Advani, Selina Luger, Richard A. Larson, Martin Tallman, Frederick Appelbaum, Mei-Jie Zhang, Khalid Bo-Subait, Hai-Lin Wang, Vijaya Raj Bhatt, Bhagirathbhai Dholaria, Mary Eapen, Mehdi Hamadani, Omer Jamy, Tim Prestidge, Michael Pulsipher, David Ritchie, David Rizzieri, Akshay Sharma, Pere Barba, Brenda M. Sandmaier, Marcos de Lima, Partow Kebriaei, Mark Litzow, Wael Saber, Daniel Weisdorf

**Affiliations:** 1 University of California, San Diego Medical Center, La Jolla, CA USA; 2 University of Chicago Medicine, Chicago, IL USA; 3 Cleveland Clinic, Taussig Cancer Institute, Cleveland, OH USA; 4 Abramson Cancer Center, University of Pennsylvania Medical Center, Philadelphia, PA USA; 5 Leukemia Service, Department of Medicine, Memorial Sloan Kettering Cancer Center, New York, NY USA; 6 Fred Hutchinson Cancer Research Center, Seattle, WA USA; 7 Center for International Blood and Marrow Transplant Research, Department of Medicine, Medical College of Wisconsin, Milwaukee, WI USA; 8 Division of Biostatistics, Institute for Health and Society, Medical College of Wisconsin, Milwaukee, WI USA; 9 The Fred and Pamela Buffett Cancer Center, University of Nebraska Medical Center, Omaha, NE USA; 10 Vanderbilt University Medical Center, Nashville, TN USA; 11 BMT and Cellular Therapy Program, Department of Medicine, Medical College of Wisconsin, Milwaukee, WI USA; 12 University of Alabama at Birmingham, Birmingham, AL USA; 13 Blood and Cancer Centre, Starship Children’s Hospital, Auckland, New Zealand; 14 Section of Transplantation and Cellular Therapy, Children’s Hospital Los Angeles Cancer and Blood Disease Institute, USC Keck School of Medicine, Los Angeles, CA USA; 15 Peter MacCallum Cancer Centre and Royal Melbourne Hospital, Melbourne, Vic Australia; 16 Division of Hematologic Malignancies and Cellular Therapy, Duke University, Durham, NC USA; 17 Department of Bone Marrow Transplantation and Cellular Therapy, St. Jude Children’s Research Hospital, Memphis, TN USA; 18 Vall Hebron University Hospital—Universitat Autonoma de Barcelona, Barcelona, Spain; 19 Division of Medical Oncology, University of Washington, Seattle, WA USA; 20 Clinical Research Division, Fred Hutchinson Cancer Research Center, Seattle, WA USA; 21 Seidman Cancer Center, University Hospitals Case Medical Center, Cleveland, OH USA; 22 Department of Stem Cell Transplantation, Division of Cancer Medicine, The University of Texas MD Anderson Cancer Center, Houston, TX USA; 23 Division of Hematology and Transplant Center, Mayo Clinic, Rochester, MN USA; 24 Division of Hematology, Oncology and Transplantation, Department of Medicine, University of Minnesota, Minneapolis, MN USA

**Keywords:** Acute lymphocytic leukaemia, Acute lymphocytic leukaemia

## Abstract

Optimal post-remission therapy for adolescents and young adults (AYAs) with Ph-negative acute lymphoblastic leukemia (ALL) in first complete remission (CR1) is not established. We compared overall survival (OS), disease-free survival (DFS), relapse, and non-relapse mortality (NRM) for patients receiving post-remission therapy on CALGB 10403 to a cohort undergoing myeloablative (MA) allogeneic hematopoietic cell transplantation (HCT) in CR1. In univariate analysis, OS was superior with chemotherapy compared to MA allogeneic HCT (3-year OS 77% vs. 53%, *P* < 0.001). In multivariate analysis, allogeneic HCT showed inferior OS (HR 2.00, 95% CI 1.5–2.66, *P* < 0.001), inferior DFS (HR 1.62, 95% CI 1.25–2.12, *P* < 0.001), and increased NRM (HR 5.41, 95% CI 3.23–9.06, *P* < 0.001) compared to chemotherapy. A higher 5-year relapse incidence was seen with chemotherapy compared to allogeneic HCT (34% vs. 23%, *P* = 0.011). Obesity was independently associated with inferior OS (HR 2.17, 95% CI 1.63–2.89, *P* < 0.001), inferior DFS (HR 1.97, 95% CI 1.51–2.57, *P* < 0.001), increased relapse (1.84, 95% CI 1.31–2.59, *P* < 0.001), and increased NRM (HR 2.10, 95% CI 1.37–3.23, *P* < 0.001). For AYA ALL patients in CR1, post-remission therapy with pediatric-style chemotherapy is superior to MA allogeneic HCT for OS, DFS, and NRM.

## Introduction

The optimal post-remission therapy for AYAs with Ph-negative ALL in CR1 is not known in the era of pediatric-inspired chemotherapy regimens with more intensive post-remission therapy than traditional adult regimens. Previous studies suggested superiority of post-remission therapy with allogeneic HCT over chemotherapy. MRC UKALLXII/E2993 compared traditional adult chemotherapy or autologous HCT to MA allogeneic HCT in patients aged 15–59 years with Ph-negative ALL. Overall survival (OS) was improved in standard-risk ALL patients with a donor, primarily due to more relapse in the no donor group [1]. However, individual studies in adults comparing allogeneic HCT to chemotherapy alone have not shown a clear benefit of allogeneic HCT although meta-analyses support allogeneic HCT in CR1 [2, 3]. A meta-analysis of 13 trials comparing allogeneic HCT to chemotherapy with or without autologous HCT concluded that the benefit of allogeneic HCT for patients with Ph-negative ALL in CR1 was limited to those under the age of 35 [2], the age group now commonly treated with pediatric-style chemotherapy. In this metanalysis, which included the UKALLXII/E2993 study, 5-year OS for patients <35 years of age was 53.7%, lower than the survival typically seen with pediatric-inspired chemotherapy in the AYA population [2, 4–8].

Pediatric-style chemotherapy may improve outcomes for AYAs with Ph-negative ALL [4–8]. A meta-analysis of 11 trials showed superior OS, event-free survival (EFS), and relapse rates in AYAs treated with pediatric-inspired chemotherapy relative to conventional adult chemotherapy [9]. The DFCI Adult ALL Consortium reported on 74 patients aged 18–50 years with Ph-negative ALL treated with pediatric-style chemotherapy [10]. A 4-year EFS of 62% was observed. Seftel et al. compared the DFCI regimen to MA allogeneic HCT outcomes using data from the Center for International Blood and Marrow Transplant Research (CIBMTR) [11]. Relapse rates were similar between the two groups but pediatric-style chemotherapy was superior to allogeneic HCT for 4-year OS and DFS, due primarily to lower rates of NRM with chemotherapy. In multivariate analysis, allogeneic HCT was the only factor associated with inferior OS (HR 2.86, *P* < 0.0001).

A single-arm, prospective, US Intergroup study led by the Alliance for Clinical Trials in Oncology (CALGB 10403) studied the feasibility of applying the pediatric COG AALL0232 standard-risk arm regimen to AYAs aged 16–39 years with Ph-negative ALL [12]. The 3-year EFS was 59% with a 3-year OS of 73%, an improvement compared to young adults treated with historical adult CALGB regimens [13]. Given the large size of the study and its enrollment of patients across the US, we used it as a model population to compare outcomes of pediatric-style post-remission therapy to MA allogeneic HCT.

A major limitation of prior studies comparing allogeneic HCT to chemotherapy is that the post-remission chemotherapy was of lower intensity compared with current pediatric-style chemotherapy regimens. Pediatric-style post-remission therapy may improve relapse rates with low NRM conceivably leading to superior survival relative to allogeneic HCT. This study was designed to compare OS, DFS, relapse, and NRM in patients aged 16–39 years with Ph-negative ALL in CR1 undergoing post-remission therapy with pediatric-inspired chemotherapy on CALGB 10403 vs. MA allogeneic HCT. Results from this study directly inform the debate on the role of allogeneic HCT for AYA patients with Ph-negative ALL in CR1.

## Methods

### Study population

The chemotherapy cohort consisted of patients receiving post-remission chemotherapy on CALGB 10403 (NCT00558519). Eligible patients from CALGB 10403 were patients aged 16–39 years with Philadelphia-chromosome/BCR-ABL1-negative ALL in CR1 who started post-remission chemotherapy. The MA allogeneic HCT cohort consisted of patients aged 16–39 years with a diagnosis of Ph-negative ALL in CR1 undergoing post-remission therapy with a MA bone marrow or peripheral blood allogeneic HCT from a matched sibling, matched related, or unrelated donor (URD) in the U.S. from 11/1/2002 through 8/31/2012 reported to the CIBMTR. Patients receiving syngeneic, haploidentical, or umbilical cord blood HCT were excluded.

### Objectives and endpoints

The start time for outcomes was the date of CR1. The primary objective was to compare OS between patients receiving post-remission therapy with MA allogeneic HCT vs. chemotherapy. Secondary objectives included DFS, relapse rate, and NRM rate between the allogeneic HCT and chemotherapy cohorts. Additional secondary objectives included determination of patient and disease factors influencing outcomes of post-remission therapy using either approach. The primary endpoint of OS was defined as the time from CR1 to the last contact date or death from any cause. Secondary endpoints included NRM defined as time to death without evidence of leukemia recurrence with relapse as the competing risk, relapse was defined as the cumulative incidence of leukemia recurrence after CR1 with NRM as a competing risk, and DFS was defined as the time from CR1 to leukemia relapse or death. Surviving patients were censored at last time reported alive and in continued remission. Allogeneic HCT cohort endpoints included acute grade II–IV graft-versus-host disease (GVHD) fulfilling the Consensus criteria and chronic GVHD defined as the occurrence of symptoms in any organ diagnostic of chronic GVHD.

### Statistical analysis

Patient-, disease-, and transplant-related factors were compared between treatment groups using the Chi-square test for categorical variables and the Wilcoxon two sample test for continuous variables. Comparing outcomes in the transplant and chemotherapy groups required adjustment for two sources of bias: differences in time to treatment/time to transplant and differences in baseline characteristics of patients. Starting from CR1, left-truncated analysis methods were used to address the first potential bias, that patients must survive in CR1 a sufficient length of time until transplant. At each time point (*t*) in this model, the risk set in the chemotherapy cohort consists of all patients still under study, while the risk set in the transplant cohort includes only those whose waiting time to transplant was less than the current study time point (*t*). The probabilities of DFS and OS were calculated using the left-truncated Kaplan–Meier estimator. Probabilities of NRM and relapse were generated using left-truncated cumulative incidence estimates to account for competing risks. The univariate probabilities of relapse and NRM were compared using the Gray’s test [14]. To adjust for the differences in baseline characteristics, left-truncated Cox proportional hazards regression was used to compare the two treatment groups. Multivariate analysis factors included age, gender, race, body mass index, Karnofsky performance status, ALL immunophenotype, white blood cell count at diagnosis, cytogenetic risk group, extramedullary disease at diagnosis, and time to documentation of CR1 (Table 1). The assumption of proportional hazards for each factor in the Cox model was tested using time-dependent covariates. When the test indicated differential effects over time (nonproportional hazards), models were constructed breaking the post-transplant time course into two periods, using the maximized partial likelihood method to find the most appropriate breakpoint. A backward stepwise model selection approach was used to identify all significant risk factors. Each step of model building contained the main effect for treatment groups. Factors, which were significant at a 5% level, were kept in the final model. The potential interactions between main effect and all significant risk factors were tested. Adjusted probabilities of DFS and OS were generated from the final Cox models stratified on treatment and weighted averages of covariate values using the pooled sample proportion as the weight function. These adjusted probabilities estimate likelihood of outcomes in populations with similar prognostic factors. Chemotherapy patients who received a transplant during CR1 were censored for OS at time of transplant (*N* = 20). HCT-related variables were not analyzed in the multivariate since they are not relevant in the chemotherapy cohort. The HCT cohort included a planned sub-analysis of donor effect evaluating HLA-identical siblings vs. well-matched (8/8) URD or partially matched (7/8) URD and no differences were seen in outcomes between the groups. Acute and chronic GVHD was included following HCT as time-dependent covariates.

**Table 1:** Population characteristics.

Characteristic	CIBMTR HCT	CALGB 10403	*P* value
No. of patients	217	263	
No. of centers	72	60	
Follow-up—median (min–max)	98 (24–173)	65 (1–109)	
**Age at CR1, Years**—no. (%) 0.08^a^			
Median (min–max)	27 (16–40)	24 (17–39)	
16–20	49 (23)	66 (25)	
21–29	94 (43)	132 (50)	
30–39	74 (34)	65 (25)	
** Gender**—no. (%)			0.51^a^
Male	140 (65)	162 (62)	
Female	77 (35)	101 (38)	
** Race**—no. (%)			<0.001^b^
Caucasian	193 (89)	197 (75)	
Other	24 (11)	41 (16)	
Unknown/missing	0	25 (10)	
** BMI**—no. (%)			0.55^a^
Not obese, BMI < 30	154 (71)	180 (68)	
Obese, BMI ≥ 30	63 (29)	83 (32)	
**Karnofsky performance score**—no. (%)			<0.001^b^
<80	12 (6)	23 (9)	
≥80	192 (88)	240 (91)	
Missing	13 (6)	0	
** Immunophenotype**—no. (%)			0.09^a^
T-cell	43 (20)	65 (25)	
B-cell	169 (78)	197 (75)	
Unspecified	5 (2)	1 (0)	
**White blood count at diagnosis** (×10^9^)—no. (%)			0.05^a^
B-cell, <30	119 (55)	157 (60)	
B-cell, >30	50 (23)	40 (15)	
T-cell, <100	36 (17)	48 (18)	
T-cell, >100	7 (3)	17 (6)	
Unspecified, <30	1 (0)	0	
Unspecified, 30–100	3 (1)	0	
Unspecified, >100	1 (0)	0	
Missing	0	1 (0)	
**Cytogenetics**—no. (%)			<0.001^a^
Normal	88 (41)	57 (22)	
Poor	35 (16)	63 (24)	
Other	68 (31)	39 (15)	
Missing	26 (12)	104 (40)	
**Extramedullary disease at diagnosi**s—no. (%)			<0.001^a^
No	176 (81)	137 (52)	
Yes	41 (19)	126 (48)	
CNS envolvement	11 (5)	27 (10)	
**Time to achieve CR1,** weeks—no. (%)			<0.001^a^
Median (min–max)	7.6 (1.3–56)	4.4 (2.3–14.4)	
≤4 weeks	35 (16)	25 (10)	
4–8 weeks	82 (38)	234 (89)	
>8 weeks	100 (46)	4 (2)	
**Transplant characteristics**			
Time from CR1 to HCT, months—no. (%)			
Median (min–max)	3.3 (0.2–18.8)		
<6 months	186 (86)		
6–12 months	30 (14)		
>12 months	1 (0)		
TBI given for conditioning—no. (%)	199 (92)		
Donor type—no. (%)			
HLA-identical sibling	75 (35)		
Other related	4 (2)		
Well-matched unrelated	93 (43)		
Partially matched unrelated	39 (18)		
Mis-matched unrelated	6 (3)		
Donor age, years—median (min–max)	34 (19–59)		
Donor/recipient gender—no. (%)			
F/M	49 (23)		
Others	168 (77)		
Donor/recipient CMV match—no. (%)			
+/−	71 (33)		
−/+	57 (26)		
−/−	34 (16)		
+/+	52 (24)		
Missing	3 (1)		
Graft type—no. (%)			
Bone marrow	58 (27)		
Peripheral blood	159 (73)		
Year of transplant—no. (%)			
2002–2005	93 (43)		
2006–2008	60 (28)		
2009–2012	64 (29)		
GVHD prophylaxis—no. (%)			
Ex-vivo or CD34 selection	10 (5)		
Tac + MTX ± others	109 (50)		
CSA + MTX ± others	42 (19)		
Others	52 (24)		
Missing	4 (2)		
T-cell depletion (ATG/Alemtuzumab)—no. (%)	45 (21)		

Bold denotes variables included in regression models. Poor: complex (≥3 abnormalities), *t*(9;22), *t*(4;11), *t*(8;14), *t*(14;18), hypodiploid (<46); normal: no abnormality; other: any abnormality not in poor. Others:_ATG + CsA + MTX + MMF + ursodiol (*n* = 1), ATG + tacrolimus ± others (*n* = 7), corticosteroid + CsA ± others (*n* = 3), cortisosteroid + tacroimus ± others (*n* = 5), CsA + tacrolimus (*n* = 1), CsA + MMF (*n* = 3), CsA + MTX + MMF (*n* = 1), tacrolimus (*n* = 3), tacrolimus + sirolimus ± others (*n* = 15), tacrolimus + MMF ± others (*n* = 10), MMF + sirolimus (*n* = 1), MTX (*n* = 2).

^a^Hypothesis testing: Pearson chi-square test.

^b^Hypothesis testing: Fisher exact test.

## Results

### Patient populations

The chemotherapy cohort consisted of 263 patients treated at 60 centers with a median age of 24 years (range 17–39) and median follow up of 65 months (range 1–109). The MA allogeneic HCT cohort consisted of 217 patients treated at 72 centers with a median age of 27 years (range 16–40) with a median follow up of 98 months (range 23–179). Significant differences between the chemotherapy and allogeneic HCT cohorts included race (Caucasian, 75% vs. 89%), Karnofsky performance score (≥80, 91% vs. 88%), white blood cell count at diagnosis, cytogenetics, extramedullary disease at diagnosis (present, 48% vs. 19%), and weeks from diagnosis to documentation of CR1 (median 4.4 vs. 7.6 weeks). A large majority of patients in the allogeneic HCT cohort patients received TBI-based conditioning (92%) from a well-matched related or URD (79%) within 6 months of documented CR1 (86%). Graft source was peripheral blood for 73% of transplants. GVHD prophylaxis was principally calcineurin inhibitor with methotrexate based (70%) with in vivo T-cell depletion used in 21% of transplants. Population characteristics are summarized in Table 1.

### Outcomes in univariate analysis

**Fig. 1:**
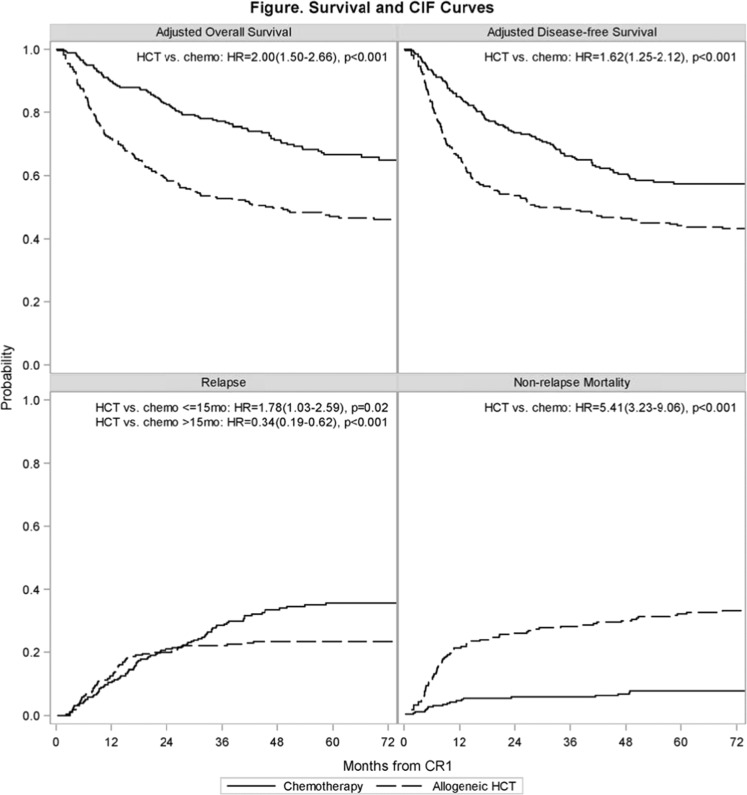
Overall survival, disease-free survival, relapse, and non-relapse mortality from first complete remission (CR1) of chemotherapy (chemo) and allogeneic HCT (HCT) cohorts. Upper left, adjusted overall survival; upper right, adjusted disease-free survival; lower left, cumulative incidence of relapse; lower right, cumulative incidence of non-relapse mortality.

In univariate analysis, no differences in OS, relapse, or NRM, were observed between allogeneic HCT and chemotherapy at 100 days after the initiation of post-remission therapy. At 3 and 5 years, OS was superior with chemotherapy compared to allogeneic HCT (3-year OS 77% vs. 53%, *P* < 0.001, 5-year OS 66% vs. 47%, *P* < 0.001). Also, 3- and 5-year DFS was superior with chemotherapy compared to allogeneic HCT (3-year DFS 68% vs. 50%, *P* < 0.001, 5-year DFS 58% vs. 44%, *P* = 0.004). Non-relapse mortality was lower with chemotherapy compared to allogeneic HCT (3-year NRM 6% vs. 24%, *P* = < 0.001. 5-year NRM 8% vs. 29%, *P* < 0.001). Cumulative relapse at 5 years was significantly higher in the chemotherapy cohort relative to the allogeneic HCT cohort (5-year relapse incidence 34% vs. 23%, *P* = 0.011). Univariate outcomes are summarized in Table 2 and Fig. 1.

**Table 2:** Univariate estimates for allogeneic HCT vs. chemotherapy.

Outcome	Myeloablative allogeneic HCT (CIBMTR)		Pediatric chemotherapy (CALGB 10403)		
	*N*	Probability, % (95% CI)	*N*	Probability, % (95% CI)	*P*
Overall survival	217		263		<0.001
100-day		95 (88–99)%		99 (98–100)%	0.090
1-year		72 (65–79)%		91 (87–94)%	<0.001
3-year		53 (46–60)%		77 (71–82)%	<0.001
5-year		47 (40–54)%		66 (60–72)%	<0.001
Relapse	215		256		0.016
100-day		0 (0–2)%		0 (0–2)%	0.909
1-year		11 (7–16)%		10 (7–15)%	0.803
3-year		21 (16–27)%		26 (21–32)%	0.216
5-year		23 (17–29)%		34 (28–40)%	0.011
Non-relapse mortality	215		256		<0.001
100-day		1 (0–3)%		1 (0–2)%	0.536
1-year		17 (12–22)%		4 (2–7)%	<0.001
3-year		24 (19–30)%		6 (3–9)%	<0.001
5-year		29 (23–35)%		8 (5–12)%	<0.001
Disease-free survival	215		256		<0.001
100-day		95 (88–99)%		99 (97–100)%	0.133
1-year		66 (59–73)%		85 (80–89)%	<0.001
3-year		50 (43–57)%		68 (62–73)%	<0.001
5-year		44 (38–51)%		58 (52–65)%	0.004

### Multivariate outcomes by treatment modality

For the entire study population (*N* = 480), OS was worse with allogeneic HCT (HR 2.00, 95% CI 1.5–2.66, *P* < 0.001) and obesity (BMI ≥ 30 kg/m^2^, HR 2.17, 95% CI 1.63–2.89, *P* < 0.001). Post-remission therapy with allogeneic HCT led to inferior DFS (HR 1.62, 95% CI 1.25–2.12, *P* < 0.001) and higher risk of NRM (HR 5.41, 95% CI 3.23–9.06, *P* < 0.001). In the early post-remission period (≤15 months after CR1), relapse was more likely with allogeneic HCT (HR 1.78, 95% CI 1.10–2.88, *P* = 0.02) while beyond 15 months after CR1 relapse was more likely in the chemotherapy cohort (HR 0.34, 95% CI 0.19–0.62, *P* < 0.001). In addition to worse OS, obesity was associated with inferior DFS (HR 1.97, 95% CI 1.51–2.57, *P* < 0.001), more relapse (HR 1.84, 95% CI 1.31–2.59, *P* < 0.001), and more NRM (HR 2.10, 95% CI 1.37–3.23, *P* < 0.001). We included a planned multivariate sub-analysis comparing outcomes of HLA-identical siblings and well-matched (8/8) URDs to the chemotherapy cohort but findings were similar to the complete transplant cohort. Multivariate outcomes are summarized in Table 3. In addition, multivariate analysis evaluating outcomes by cytogenetic risk (standard vs. poor) and HCT time period (2002–2006 vs. 2007–2012) showed similar results to the full study population (Supplementary Tables 1 and 2).

**Table 3:** Multivariate analysis.

Outcomes	*N*	HR (95% CI)	*P* value
**Overall survival**			
Main effect			
Chemotherapy	261	Reference	
Allogeneic HCT	217	2.00 (1.50–2.66)	<0.001
Body mass index (kg/m^2^)			
<30	332	Reference	
≥30	146	2.17 (1.63–2.89)	<0.001
**Disease-free survival**			
Main effect			
Chemotherapy	261	Reference	
Allogeneic HCT	215	1.62 (1.25–2.12)	<0.001
Body mass index (kg/m^2^)			
<30	331	Reference	
≥30	145	1.97 (1.51–2.57)	<0.001
**Relapse**			
Allogeneic HCT vs. chemotherapy ≤ 15 months after CR1		1.78 (1.10–2.88)	0.02
Allogeneic HCT vs. chemotherapy > 15 months after CR1		0.34 (0.19–0.62)	<0.001
Body mass index (kg/m^2^)			
<30	331	Reference	
≥30	145	1.84 (1.31–2.59)	<0.001
**Non-relapse mortality**			
Main effect			
Chemotherapy	261	Reference	
Allogeneic HCT	215	5.41 (3.23–9.06)	<0.001
Body mass index (kg/m^2^)			
<30	331	Reference	
≥30	145	2.10 (1.37–3.23)	<0.001

## Discussion

Allogeneic HCT is indicated for Ph-negative ALL in CR1 based on trials using donor vs. no donor comparisons. UKALLXII/ECOG2993 showed improved survival with allogeneic HCT for adults aged 15–54 years with Ph-negative ALL when compared to chemotherapy or autologous HCT (5-year OS 54% vs. 44%, *P* = 0.007) [1]. Gupta et al. found in a meta-analysis of 13 studies that allogeneic HCT for Ph-negative ALL benefited patients under age 35 years relative to traditional adult chemotherapy (Odd ratio of death = 0.79; 95% CI, 0.70–0.90, *P* = 0.0003) [2]. However, pediatric-style regimens with more intensive post-remission therapy appear to have improved outcomes for AYA patients with Ph-negative ALL in retrospective comparisons [4–9]. A retrospective analysis of GRAALL2003/2005 studies showed that, for adults aged 15–55 years with high-risk Ph-negative ALL, allogeneic HCT demonstrated equivalent survival to pediatric-style chemotherapy in patients MRD-negative after induction and a survival benefit for patients MRD-positive after induction [15]. The finding suggested that allogeneic HCT is a safe and effective option for all high-risk ALL patients regardless of MRD status.

To help clarify the role of allogeneic HCT in AYAs with Ph-negative ALL in CR1 treated with pediatric-style chemotherapy, Seftel et al. compared 108 adults 18–50 years of age treated with the Dana-Farber Consortium pediatric-style regimen at 13 centers in the northeastern US and eastern Canada to a CIBMTR allogeneic HCT cohort [11]. No difference in relapse was observed but higher NRM, lower DFS, and lower OS was seen with allogeneic HCT. Notably, allogeneic HCT was the only multivariate factor predictive of shorter OS (HR 3.12 (1.99–4.90), *P* < 0.0001). Limitations of the study included a chemotherapy cohort with small numbers of patients from a select and geographically limited group of centers compared to the CIBMTR cohort. As such, the generalizability of the findings is unclear but support deferring allogeneic HCT for relapse in AYAs receiving pediatric-style post-remission therapy.

CALGB 10403 was a national, single-arm, Phase II study of the feasibility of treating patients aged 16–39 years with Ph-negative ALL with the standard-risk arm of the COG AALL0232 regimen [12, 13]. The study analyzed 295 patients at 60 centers across the United States, among which 263 achieved complete remission after initial treatment. Comparing this patient cohort to a contemporary CIBMTR allogeneic HCT cohort, we found improved OS with pediatric-style post-remission chemotherapy relative to MA allogeneic HCT with an absolute 5-year survival improvement of 19%. Although overall relapse was higher with pediatric-style chemotherapy, the high NRM with allogeneic HCT had a large negative impact on OS. In multivariate analysis, allogeneic HCT was significantly associated with reduced OS, reduced DFS, and increased NRM. Differences in relapse between the cohorts seen in our study versus the Seftel et al. study [11] may be related to differences in chemotherapy regimens, patient compliance, patient eligibility, and/or statistical power. In contrast to the high-risk Ph-negative ALL patients treated on the GRAALL2003/2005, patients treated with CALGB 10403 consolidation had significantly improved survival relative to allogeneic HCT patients. The different patient populations or possibly the different post-remission regimens may explain the superiority of chemotherapy over allogeneic HCT seen in our study.

In pediatric patients with ALL receiving chemotherapy, obesity is associated with increased NRM, increased relapse, and decreased OS [16–22], but a negative effect of obesity has not been routinely seen in adults [23]. CALGB 10403 found that obese AYA patients with ALL had significantly reduced DFS compared with nonobese patients [13]. We found that obesity was associated with decreased OS and DFS in this study and the effect was seen in both the chemotherapy and allogeneic HCT cohorts. The difference was due to both increased relapse and increased NRM in obese patients. The curative potential of both chemotherapy and allogeneic HCT appears to be reduced in obese AYAs with ALL. Adipocytes appear to protect ALL cells from the toxic effects of chemotherapy including daunorubicin, by sequestration and metabolism, and asparaginase, through the release of glutamine [24–29]. NRM was also higher in obese patients perhaps owing to lower tolerability of treatment due to comorbidities, high doses of therapy dosed on body-surface area, or alternative pharmacokinetics or pharmacodynamics of drugs in the obese population. Prior large studies focusing of obesity in adults undergoing allogeneic HCT for hematologic malignancies have not found a deleterious effect of obesity on OS or relapse with some studies showing increased NRM [30–34]. It is possible that the negative effect of obesity on OS and relapse seen here may be unique to ALL and the AYA population.

One limitation of this study is that minimal information on MRD in both cohorts prevented meaningful comparisons between MRD-positive and MRD-negative patients in both cohorts. As such, the role of allogeneic HCT vs. chemotherapy in MRD-positive patients could not be explored. In addition, it is possible that the allogenic HCT cohort had higher risk features not identified here, including higher rates of MRD positivity, although if true this did not translate into high relapse rates in the allogeneic HCT cohort and would not be expected to affect NRM, the primary reason for the reduced survival seen with allogeneic HCT relative to pediatric-style chemotherapy. The chemotherapy regimens received prior to allogeneic HCT in the transplant cohort were also not available and this may affect relapse rates and possibly NRM. Again, however, relapse rates were low with allogeneic HCT and superior to chemotherapy whereas NRM was substantially higher with allogeneic HCT, an outcome less likely to be impacted by prior treatment with ALL-directed chemotherapies relative to the chemotherapy cohort. Another limitation is the lack of testing for the Ph-like phenotype and IKAROS mutation/loss in the allogeneic HCT cohort. Although traditional high-risk cytogenetic features had no impact on outcomes, we were unable to assess if patients with Ph-like ALL may benefit from allogeneic HCT in CR1. Notable difference in the cohorts included extramedullary disease at diagnosis and longer time to documentation of CR1 in the allogeneic HCT cohort. Outside a formal clinical trial, documentation of extramedullary disease may be incomplete and the timing of remission assessments may vary based on treatment regimen used and post-induction marrow assessments, which in the transplant cohort were not determined by trial timepoints. Last, changes in treatment modalities, addition of new therapeutic agents, and improvements in supportive care may have changed relapse rates and NRM in both populations since 2012.

For AYAs with Ph-negative ALL in CR1, we conclude that AYAs receiving post-remission therapy with pediatric-style chemotherapy for Ph-negative ALL in CR1 should not routinely undergo allogeneic HCT. Allogeneic HCT is warranted for patients refractory to or relapsing after pediatric-style regimens and may be warranted for high-risk MRD-positive patients in CR1, although more study is needed. Obesity in AYAs with ALL warrants further investigation as a potentially modifiable factor that negatively impacted survival.

## Supplementary information

Supplementary Table 1

Supplementary Table 2
