# Supplementary material for: Superior survival with pediatric-style chemotherapy compared to myeloablative allogeneic hematopoietic cell transplantation in older adolescents and young adults with Ph-negative acute lymphoblastic leukemia in first complete remission: analysis from CALGB 10403 and the CIBMTR
Source: Leukemia. 2021 Mar 30;35(7):2076–85. doi: 10.1038/s41375-021-01213-5 (PMC8257494; doi:10.1038/s41375-021-01213-5)
Supplement: Supplementary file 1 — Supplementary Table 1 [file 41375_2021_1213_MOESM1_ESM.docx]

**Supplementary Table 1: Multivariate sensitivity analysis for cytogenetic risk**

|  | **Standard cytogenetic risk** | | | **Poor cytogenetic risk** | | |
| --- | --- | --- | --- | --- | --- | --- |
| **Covariates** | **N** | **HR (95% CI)** | **p-value** | **N** | **HR (95% CI)** | **p-value** |
| **Overall Survival** |  |  |  |  |  |  |
| Main Effect |  |  |  |  |  |  |
| Chemotherapy | 96 | Reference |  | 63 | Reference |  |
| Allogeneic HCT | 156 | 1.92 (1.28-2.88) | 0.001 | 35 | 1.90 (0.99-3.67) | 0.06 |
| **Disease Free Survival** |  |  |  |  |  |  |
| Main Effect |  |  |  |  |  |  |
| Chemotherapy | 93 | Reference |  | 63 | Reference |  |
| Allogeneic HCT | 155 | 1.59 (1.10-2.31) | 0.01 | 35 | 1.88 (1.00-3.55) | 0.05 |
| **Relapse** |  |  |  |  |  |  |
| HCT vs. chemo <=15 months after CR1 |  | 2.16 (1.04-4.47) | 0.04 |  | 1.74 (0.54-5.56) | 0.35 |
| HCT vs. chemo >15 months after CR1 |  | 0.31 (0.15-0.63) | 0.001 |  | 0.44 (0.10-2.06) | 0.30 |
| **Non-relapse Mortality** |  |  |  |  |  |  |
| Main Effect |  |  |  |  |  |  |
| Chemotherapy | 93 | Reference |  | 63 | Reference |  |
| Allogeneic HCT | 155 | 6.47 (2.75-15.2) | < 0.001 | 35 | 4.78 (1.65-13.9) | 0.004 |
