# Supplementary material for: Superior survival with pediatric-style chemotherapy compared to myeloablative allogeneic hematopoietic cell transplantation in older adolescents and young adults with Ph-negative acute lymphoblastic leukemia in first complete remission: analysis from CALGB 10403 and the CIBMTR
Source: Leukemia. 2021 Mar 30;35(7):2076–85. doi: 10.1038/s41375-021-01213-5 (PMC8257494; doi:10.1038/s41375-021-01213-5)
Supplement: Supplementary file 2 — Supplementary Table 2 [file 41375_2021_1213_MOESM2_ESM.docx]

**Supplementary Table 2: Multivariate sensitivity analysis for time period of allogeneic HCT**

|  | **HCT in 2002-2006** | | | **HCT in 2007-2012** | | |
| --- | --- | --- | --- | --- | --- | --- |
| **Covariates** | **N** | **HR (95% CI)** | **p-value** | **N** | **HR (95% CI)** | **p-value** |
| **Overall Survival** |  |  |  |  |  |  |
| Main Effect |  |  |  |  |  |  |
| Chemotherapy | 261 | Reference |  | 261 | Reference |  |
| Allogeneic HCT | 93 | 2.32 (1.63-3.29) | < 0.001 | 124 | 1.74 (1.25-2.44) | 0.001 |
| **Disease Free Survival** |  |  |  |  |  |  |
| Main Effect |  |  |  |  |  |  |
| Chemotherapy | 254 | Reference |  | 254 | Reference |  |
| Allogeneic HCT | 91 | 1.89 (1.35-2.65) | < 0.001 | 124 | 1.56 (1.14-2.15) | 0.006 |
| **Relapse** |  |  |  |  |  |  |
| HCT vs. chemo <=15 months after CR1 |  | 2.05 (1.12-3.77) | 0.02 |  | 1.63 (0.92-2.90) | 0.10 |
| HCT vs. chemo >15 months after CR1 |  | 0.26 (0.09-0.72) | 0.009 |  | 0.45 (0.23-0.89) | 0.02 |
| **Non-relapse Mortality** |  |  |  |  |  |  |
| Main Effect |  |  |  |  |  |  |
| Chemotherapy | 254 | Reference |  | 254 | Reference |  |
| Allogeneic HCT | 91 | 6.34 (3.57-11.3) | < 0.001 | 124 | 4.62 (2.61-8.17) | < 0.001 |
